# Comparison of CT volumetry versus nuclear renography for predicting remaining kidney function after uninephrectomy in living kidney donors

**DOI:** 10.1038/s41598-022-09187-9

**Published:** 2022-03-24

**Authors:** Sang Hun Eum, Hanbi Lee, Eun Jeong Ko, Hyuk Jin Cho, Chul Woo Yang, Byung Ha Chung

**Affiliations:** 1 Division of Nephrology, Department of Internal Medicine, Seoul St. Mary’s Hospital, College of Medicine, The Catholic University of Korea, 222 Banpo-Daero, Seocho-Ku, 137-040 Seoul, Republic of Korea; 2 Transplant Research Center, Convergent Research Consortium for Immunologic Disease, Seoul St. Mary’s Hospital, College of Medicine, The Catholic University of Korea, Seoul, Republic of Korea; 3 Department of Urology, Seoul St. Mary’s Hospital, The Catholic University of Korea, Seoul, Republic of Korea

**Keywords:** Medical research, Nephrology

## Abstract

Computed tomography (CT) and nuclear renography are used to determine kidney procurement in living kidney donors (LKDs). The present study investigated which modality better predicts kidney function after donation. This study included 835 LKDs and they were divided into two subgroups based on whether the left–right dominance of kidney volume was concordant with kidney function (concordant group) or not (discordant group). The predictive value for post-donation kidney function between the two imaging modalities was compared at 1 month, 6 months, and > 1 year in total cohort, concordant, and discordant groups. Split kidney function (SKF) measured by both modalities showed significant correlation with each other at baseline. SKFs of remaining kidney measured using both modalities before donation showed significant correlation with eGFR (estimated glomerular filtration rate) after donation in the total cohort group and two subgroups, respectively. CT volumetry was superior to nuclear renography for predicting post-donation kidney function in the total cohort group and both subgroups. In the discordant subgroup, a higher tendency of kidney function recovery was observed when kidney procurement was determined based on CT volumetry. In conclusion, CT volumetry is preferred when determining procurement strategy especially when discordance is found between the two imaging modalities.

## Introduction

Kidney transplantation (KT) is accepted as the treatment of choice for patients with end-stage kidney disease (ESKD) because it provides better patient survival and quality of life than hemodialysis or peritoneal dialysis^1,2^. In addition, living donor KT (LDKT) showed superior allograft and patient survival than deceased donor KT (DDKT), therefore, LDKT is recommended for ESKD patients when a potential donor is available^3^. However, issues of donor safety after kidney uninephrectomy have been consistently raised. In some previous reports, kidney donation was suggested associated with the progression to chronic kidney disease and even mortality^4,5^. Consequently, a thorough baseline evaluation of the kidney donor candidate is essential^6,7^.

The serum creatinine-based equation for estimated glomerular filtration rate (eGFR) is recommended for the initial assessment of donor kidney function^7^. However, because the relative contribution of each kidney to total kidney function cannot be assessed using the eGFR, additional imaging techniques are required. In clinical practice, technetium-99 m diethylenetriamine penta-acetic acid (DTPA) nuclear renography and computed tomography (CT) scan with volumetry have been widely utilized to evaluate split kidney function (SKF) and volume. Nuclear renography accurately determines SKF of an individual kidney^8^. Although volume and function are distinct properties, CT volumetry also provides a reliable estimate of SKF compared with nuclear renography^9–11^. Reportedly, both imaging modalities are useful for predicting the post-donation function of the remaining kidney and CT volumetry was shown superior to nuclear renography in a few studies^11–14^. However, the studies were conducted with a relatively small group of donors and research on which modality is more reliable when CT volumetric and nuclear renographic measurements of split kidney function show discordance has not been conducted.

Based on the above-mentioned background, in the present study, CT volumetry and nuclear renography for the prediction of post-donation kidney function were compared using a well-established large kidney donor cohort and a more reliable imaging modality for predicting kidney outcome, especially in case of discordance, was determined. Based on this investigation, clinical guidance for determining kidney procurement with preserved-kidney-function-first consideration was suggested.

## Results

### Baseline characteristics of the study cohort

Baseline donor characteristics are presented in Table 1. Mean age at kidney donation was 43.76 ± 12.07 years and 56.41% of the donors were female. Average eGFR was 100.01 ± 13.94 mL/min/1.73 m^2^ and measured glomerular filtration rate (mGFR) was 107.16 ± 17.39 mL/min. In CT volumetry, the left kidney was significantly larger than the right kidney (right kidney, 99.85 ± 14.90 mL/min/m^2^; left kidney, 104.83 ± 16.50 mL/min/m^2^, p < 0.001). In nuclear renography, mGFR did not differ between the left and right kidney (right kidney 31.53 ± 5.43 mL/min/m^2^; left kidney 31.39 ± 5.76 mL/min/m^2^, p = 0.357). In 83.96% of total kidney donors, left nephrectomy was performed. The mean value of the remaining kidney volume was 101.68 ± 15.56 cm^3^/m^2^ and mean mGFR value of the remaining kidney was 32.35 ± 5.49 mL/min/m^2^. When CT volumetry and nuclear renography were performed, discordant results were observed in 326 subjects (39%). Table 2a shows demographic and clinical characteristics of concordant and discordant groups. All parameters showed no significant difference (all p > 0.05). Among 326 cases in the discordant group, the kidney with higher mGFR was preserved in 260 donors (nuclear renography preferred group), and in 66 donors, the kidney with larger volume was preserved (CT volumetry preferred group). The baseline characteristics showed average remaining kidney volume was higher in the CT volumetry preferred group and average remaining kidney mGFR was higher in the nuclear renography preferred group. Baseline eGFR was higher in CT volumetry preferred group (Table 2b).

**Table 1:** Demographic and clinical characteristics of the donors.

Number of studied donors	835		
Age at kidney donation (years)	43.76 ± 12.07		
Sex (female)	471 (56.41)		
BMI (kg/m^2^)	23.91 ± 3.23		
Living donor type (related)	513 (61.44)		
Donated kidney (Lt.)	701 (83.96)		
Remaining kidney volume/BSA (cm^3^/m^2^)	101.68 ± 15.56		
Remaining kidney mGFR/BSA (mL/min/m^2^)	32.35 ± 5.49		
Baseline serum creatinine (mg/dL)	0.81 ± 0.16		
eGFR (CKD-EPI) (mL/min/1.73m^2^)	100.01 ± 13.94		
mGFR (Plasma method DTPA) (mL/min)	107.16 ± 17.39		

	Right kidney	Left kidney	P value
Kidney volume/BSA (cm^3^/m^2^)	99.85 ± 14.90	104.83 ± 16.50	< 0.001
mGFR/BSA (mL/min/m^2^)	31.53 ± 5.43	31.39 ± 5.76	0.357

Values are mean ± standard deviation or frequency (%).

*BMI* body mass index, *DTPA* diethylenetriamine penta-acetic acid, *GFR* glomerular filtration rate, *eGFR* estimated glomerular filtration rate, *mGFR* measured glomerular filtration rate.

**Table 2:** Demographic and Clinical Characteristics comparison between (a) concordant and discordant groups, (b) CT volumetry preferred and nuclear renography preferred groups.

(a)	Concordant group (n = 509)	Discordant group (n = 326)	P value
Remaining kidney volume/BSA (cm^3^/m^2^)	102.11 ± 16.02	101.01 ± 14.82	0.320
Remaining kidney mGFR/BSA (mL/min/m^2^)	32.34 ± 5.56	32.36 ± 5.39	0.974
Age at kidney donation (years)	43.90 ± 11.91	43.56 ± 12.32	0.694
Sex (female)	282/509 (55.40%)	189/326 (57.98%)	0.465
BMI (kg/m^2^)	23.81 ± 3.24	24.08 ± 3.23	0.236
Baseline eGFR (CKD-EPI) (mL/min/1.73m^2^)	99.97 ± 14.01	100.07 ± 13.87	0.926
eGFR (CKD-EPI) at 1 month (mL/min/1.73m^2^)	65.82 ± 12.74	65.97 ± 14.10	0.871
eGFR (CKD-EPI) at 6 months (mL/min/1.73m^2^)	68.97 ± 13.82	68.78 ± 14.46	0.847

(b)	CT volumetry preferred group (n = 66)	Nuclear renography preferred group (n = 260)	P value
Remaining kidney volume/BSA (cm^3^/m^2^)	107.78 ± 15.80	99.30 ± 14.08	< 0.001
Remaining kidney mGFR/BSA (mL/min/m^2^)	31.08 ± 4.63	32.68 ± 5.53	0.030
Age at kidney donation (years)	41.08 ± 10.74	44.19 ± 12.63	0.067
Sex (female)	38/66 (57.58%)	151/260 (58.08%)	0.941
BMI (kg/m^2^)	24.62 ± 3.32	23.94 ± 3.20	0.129
Baseline eGFR (CKD-EPI) (mL/min/1.73m^2^)	104.12 ± 10.33	99.04 ± 14.46	0.001
eGFR (CKD-EPI) at 1 month (mL/min/1.73m^2^)	68.93 ± 11.64	65.22 ± 14.59	0.056
eGFR (CKD-EPI) at 6 months (mL/min/1.73m^2^)	71.92 ± 12.64	67.98 ± 14.81	0.031

*BMI* body mass index, *BSA* body surface area, *CT* computed tomography, *eGFR* estimated glomerular filtration rate, *mGFR* measured glomerular filtration rate.

### Intercorrelations among total kidney volume, mGFR, and eGFR at baseline

**Figure 1:**
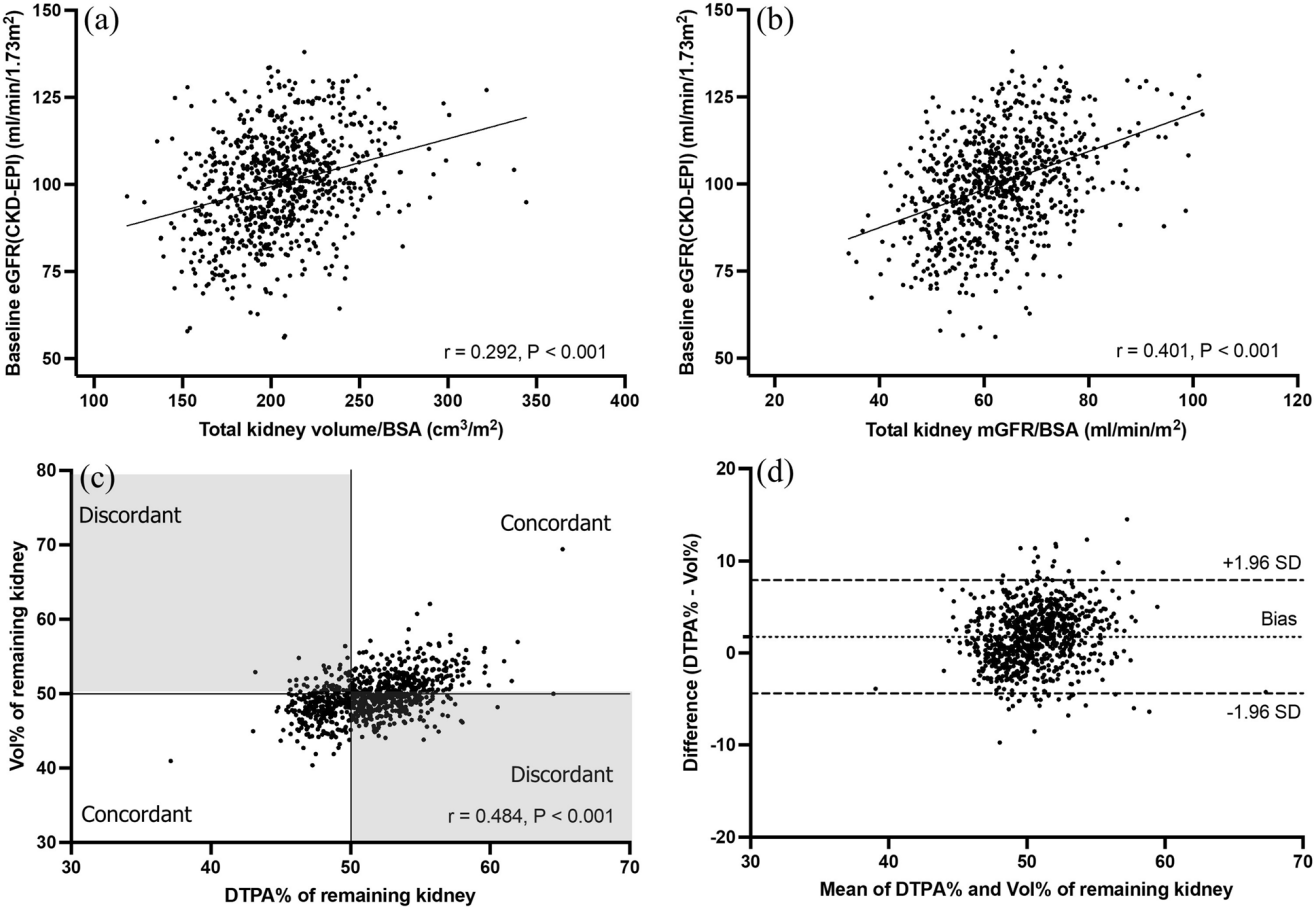
Correlations and agreement of baseline measures. (**a**) Correlation of baseline eGFR with total kidney volume/BSA. (**b**) Correlation of baseline eGFR with total kidney mGFR/BSA. (**c**) Scatter plot showing correlation of DTPA% with Vol% of remaining kidney. White fields represent concordant pairs (n = 509, 61%) and grey fields represent discordant pairs (n = 326, 39%). (**d**) Bland–Altman plot for agreement between DTPA% and Vol% of remaining kidney. Dotted line indicates the mean value of differences (y = 1.76) and dashed line indicates 95% CI for difference score data set (± 1.96*SD). *BSA* body surface area, *CI* confidence interval, *DTPA* diethylenetriamine penta-acetic acid, *eGFR* estimated glomerular filtration rate, *mGFR* measured glomerular filtration rate, *SD* standard deviation.

Both total kidney volume and total kidney mGFR significantly correlated with baseline eGFR (total kidney volume/body surface area (BSA): Pearson r = 0.292, p < 0.001; total kidney mGFR/BSA: Pearson r = 0.401, p < 0.001; Fig. 1a,b). Furthermore, total kidney volume significantly correlated with total kidney mGFR (Pearson r = 0.363, p < 0.001). Split kidney volume percent (Vol%) based on CT volumetry and split kidney mGFR percent (DTPA%) based on nuclear renography of the remaining kidney were also significantly correlated (Pearson r = 0.484, p < 0.001; Fig. 1c). The Bland–Altman plot showed the mean bias ± SD (standard deviation) between DTPA% and Vol% was 1.76 ± 3.14, and the limits of agreement were − 4.39 and 7.91, respectively, confirming that most of the differences in scores were within the 95% confidence interval (CI) of the differences (Fig. 1d). Intraclass correlation coefficient (ICC) (2,1) was 0.647 (95% CI 0.596–0.692, p < 0.001).

### Change of kidney function after uninephrectomy and its correlation with baseline SKF (Vol% or DTPA%) in the total cohort group and two subgroups

**Figure 2:**
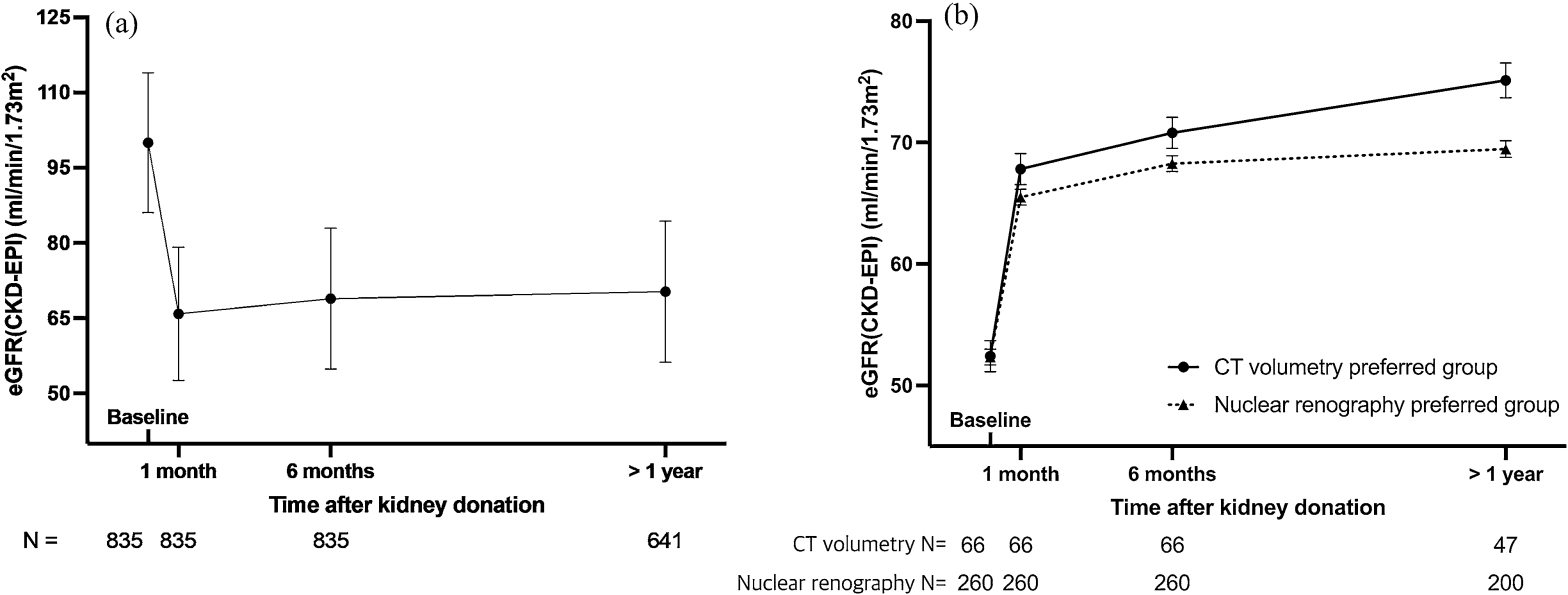
Change of eGFR after kidney donation. (**a**) eGFR (mean ± SD) change over time in the total cohort group. Baseline eGFR is total eGFR from both kidneys. (**b**) eGFR (estimated marginal mean ± SE) over time in the CT volumetry preferred group (solid line) and nuclear renography preferred group (dotted line). Estimated marginal means are derived from linear mixed model. Baseline eGFR is SKF of the remaining kidney. *CT* computed tomography, *eGFR* estimated glomerular filtration rate, *SD* standard deviation, *SE* standard error, *SKF* split kidney function.

Figure 2a shows the change of kidney function based on eGFR over time after uninephrectomy in kidney donors. The mean eGFR was 100.01 ± 13.94 mL/min/1.73 m^2^ at baseline. After kidney donation, the mean eGFR declined to 65.88 ± 13.28 mL/min/1.73 m^2^ at 1 month, recovered to 68.90 ± 14.07 mL/min/1.73 m^2^ at 6 months, and reached 70.55 ± 14.06 mL/min/1.73 m^2^ at > 1 year. SKF(DTPA%) of the remaining kidney at baseline showed significant correlation with eGFR at each time point after donation (Pearson r = 0.728 (1 month), 0.743 (6 months), 0.692 (> 1 year), all p < 0.05). SKF(Vol%) of the remaining kidney at baseline also had a significant positive correlation with eGFR at each time point after donation and correlation coefficients were higher than SKF(DTPA%) at all time points (Pearson r = 0.744 (1 month), 0.765 (6 months), 0.722 (> 1 year), all p < 0.05; Table 3). In the concordant group, SKF(Vol%) and SKF(DTPA%) showed significant positive correlations at 1 month, 6 months, and > 1 year. Similarly, significant positive correlations were found between SKF (Vol% and DTPA%) and eGFR at all time points in the discordant group. Furthermore, in both concordant and discordant groups, CT volumetry exhibited higher correlation coefficients with post-donation kidney function.

**Table 3:** Correlation and multivariable linear regression analyses of post-donation eGFR with SKF (Vol% and DTPA%).

(a) Total cohort		Correlation		Multivariable linear regression			
eGFR	Split kidney function	Pearson’s r	P value	β coefficient	P value	Relative importance	Adjusted R^2^
At 1 month (n = 835)	SKF(Vol%)	0.744	< 0.001	0.402	< 0.001	38.28%	0.605
	SKF(DTPA%)	0.728	< 0.001	0.242	< 0.001	35.46%	
At 6 months (n = 835)	SKF(Vol%)	0.765	< 0.001	0.448	< 0.001	39.56%	0.627
	SKF(DTPA%)	0.743	< 0.001	0.214	< 0.001	35.50%	
At > 1 year (n = 641)	SKF(Vol%)	0.722	< 0.001	0.472	< 0.001	40.59%	0.554
	SKF(DTPA%)	0.692	< 0.001	0.160	0.015	34.71%	

(b) Concordant group		Correlation		Multivariable linear regression			
eGFR	Split kidney function	Pearson’s r	P value	β coefficient	P value	Relative importance	Adjusted R^2^
At 1 month (n = 509)	SKF(Vol%)	0.756	< 0.001	0.391	< 0.001	36.77%	0.623
	SKF(DTPA%)	0.743	< 0.001	0.229	0.012	35.27%	
At 6 months (n = 509)	SKF(Vol%)	0.752	< 0.001	0.484	< 0.001	38.03%	0.609
	SKF(DTPA%)	0.732	< 0.001	0.133	0.149	34.71%	
At > 1 year (n = 394)	SKF(Vol%)	0.697	< 0.001	0.444	< 0.001	38.16%	0.518
	SKF(DTPA%)	0.676	< 0.001	0.126	0.267	34.65%	

(c) Discordant group		Correlation		Multivariable linear regression			
eGFR	Split kidney function	Pearson’s r	P value	β coefficient	P value	Relative importance	Adjusted R^2^
at 1 month (n = 326)	SKF(Vol%)	0.740	< 0.001	0.453	< 0.001	41.10%	0.595
	SKF(DTPA%)	0.713	< 0.001	0.259	< 0.001	35.41%	
at 6 months (n = 326)	SKF(Vol%)	0.792	< 0.001	0.480	< 0.001	42.08%	0.667
	SKF(DTPA%)	0.766	< 0.001	0.285	< 0.001	36.64%	
at > 1 year (n = 247)	SKF(Vol%)	0.773	< 0.001	0.575	< 0.001	44.19%	0.634
	SKF(DTPA%)	0.723	< 0.001	0.188	0.020	33.67%	

Covariables for multivariable linear regression analyses: donor age, sex, BMI

*BMI* body mass index, *CT* computed tomography, *DTPA* diethylenetriamine penta-acetic acid, *eGFR* estimated glomerular filtration rate.

### Multivariable linear regression analyses of donor kidney outcome

Multivariable linear regression analyses of postoperative eGFR were performed to quantify the predictive value of CT volumetry (SKF(Vol%)) and nuclear renography (SKF(DTPA%)) in the total cohort group and two subgroups (Table 3). In the total cohort group, although both imaging modalities were predictive of postoperative eGFR at 1 month, 6 months, and > 1 year, CT volumetry was more reliable than nuclear renography when adjusted for covariables (donor age, sex, and body mass index (BMI)) (1 month: β_CT_ = 0.402, β_DTPA_ = 0.242; 6 months: β_CT_ = 0.448, β_DPTA_ = 0.214; > 1 year: β_CT_ = 0.472, β_DTPA_ = 0.160, all p < 0.05). Both in the concordant and discordant groups, results were consistent with the total cohort group, showing CT volumetry was superior to nuclear renography. In the concordant group, nuclear renography did not contribute to the regression model at 6 months (β_DTPA_ = 0.133, p = 0.149) and > 1 year (β_DTPA_ = 0.126, p = 0.267). However, in Johnson’s relative weights analysis, relative importance of CT volumetry was consistently higher in the total cohort group and two subgroups.

### Comparison of kidney function recovery in the discordant group based on the procurement strategy

Based on the procurement strategy, as mentioned previously, the discordant group was divided into CT volumetry preferred group and nuclear renography preferred group. A linear mixed-effect model was used to analyze kidney function recovery. The omnibus tests showed all fixed effects were statistically significant. Figure 2b shows eGFR over time for the CT volumetry preferred and nuclear renography preferred groups. The baseline SKF did not differ between the two groups at baseline (p = 0.963). However, after controlling for other factors, interactions between time point and group indicated a higher rate of functional recovery in the CT volumetry preferred group at all time points (1 month: p = 0.071, 6 months: p = 0.071, and > 1 year: p < 0.001; Table 4).

**Table 4:** A Linear mixed-effect model for analyzing interaction of group and time with eGFR as the dependent variable.

		Estimate	SE	95% CI	P value
Intercept		79.60	4.39	70.96–88.24	< 0.001
Age		− 0.56	0.04	− 0.64–− 0.48	< 0.001
Sex (female)		5.40	1.01	3.40–7.39	< 0.001
BMI		− 0.47	0.15	− 0.77–− 0.17	0.002
**Groups**					0.030
CT volumetry Preferred group		0.07	1.44	− 2.77–2.90	0.963
Nuclear renography Preferred group		Reference			
Time-points					< 0.001
Baseline		Reference			
1 month		13.15	0.56	12.06–14.25	< 0.001
6 months		15.91	0.62	14.70–17.12	< 0.001
> 1 year		17.12	0.68	15.78–18.45	< 0.001
Group × time-points					0.004
CT volumetry preferred group	Baseline	Reference			
	1 month	2.24	1.24	− 0.19–4.68	0.071
	6 months	2.47	1.37	− 0.21–5.16	0.071
	> 1 year	5.58	1.54	2.56–8.61	< 0.001
Nuclear renography preferred group	Baseline	Reference			
	1 month	Reference			
	6 months	Reference			
	> 1 year	Reference			

*BMI* body mass index, *CI* confidence interval, *CT* computed tomography, *eGFR* estimated glomerular filtration rate, *SE* standardized error.

## Discussion

Although successful achievement of optimal graft outcome and survival for the recipient is important in LDKT, donor safety should be the primary concern. Therefore, careful evaluation of SKF for the kidney donor and meticulous decision of donor nephrectomy are essential and the better functioning kidney should stay with the donor. In the present study, both CT volumetry and nuclear renography were useful for predicting kidney function after uninephrectomy, however, CT volumetry showed superior performance especially when discordance occurred between the two imaging modalities in predicting kidney outcome.

First, the baseline characteristics, including the laterality of kidney procurement, were investigated. According to the donor evaluation protocol of our transplantation center, both CT volumetry and nuclear renography are performed for donor candidate evaluation. In general, the left kidney is preferred because the longer left renal vein may reduce operative difficulty in obtaining the donor kidney and may render the operation less demanding^15^. In the present study, 84% of the kidney donors donated the left kidney and only 16% donated the right kidney due to parenchymal, vascular, urological anatomies, or asymmetry in kidney function. The right kidney was approximately 5% smaller than the left kidney, which agrees with the literature^7^. However, functional discrepancy was not observed between the left and right kidney based on nuclear renography, indicating there can be a significant discordance between CT volumetry and nuclear renography in estimating left–right dominance of the kidneys.

Second, intercorrelations among the kidney volume, mGFR, and baseline eGFR were investigated. As reported in previous studies, a positive correlation was also found between total kidney volume and baseline eGFR^16,17^. In addition, similar positive correlation was observed between total kidney mGFR and baseline eGFR, in agreement with the results obtained by Burballa et al*.*^18^. Total kidney volume also significantly correlated with total kidney mGFR similar to the report by Halleck et al*.*^13^. Because in several previous studies kidney volume was shown a surrogate for kidney function^10,11,14^, our data comparing SKF measured using CT volumetry and nuclear renography showed good agreement between the two imaging modalities.

Next, the two imaging techniques for the prediction of post-donation eGFR were compared. Data from Barbas et al*.* and Halleck et al*.* showed superiority of CT volumetry to nuclear renography for predicting kidney function at 6 months after nephrectomy^12,13^. In addition, Patankar et al*.* and Wahba et al*.* demonstrated CT volumetric estimation of SKF is a better predictor of kidney function at 1 year after nephrectomy^11,14^. Both CT volumetry and nuclear renography showed significant correlations with post-donation kidney functions at all time points (1 month, 6 months, and > 1 year), and CT volumetry showed higher correlation coefficient values. Furthermore, multivariable linear regression was performed to compare the strength of the two imaging modalities for predicting kidney function after uninephrectomy. Because higher pre-donation BMI was consistently reported associated with unfavorable kidney function recovery and even ESKD^17,19,20^, our analyses included donor age, sex, and BMI as covariables. Results showed CT volumetry outperformed nuclear renography for predicting eGFR at 1 month, 6 months, and > 1 year after kidney donation.

The main focus in the present study was on which method is better when discordance is detected between CT volumetry and nuclear renography. Although concordance in the two measurements reinforces the decision for donor nephrectomy, discordance hampers the decision-making of clinicians. However, comparison of the ability of CT volumetry and nuclear renography for predicting kidney function after donation in discordance has not been previously investigated. Therefore, the remaining kidneys, which presented discordance, were further identified to investigate which imaging technique performed better. Consequently, in concordant and discordant subgroups, CT volumetry outperformed nuclear renography for predicting kidney function at all time points (1, 6 months, and > 1 year). These results indicate CT volumetry should be the method of choice when discordance occurs between volume and mGFR in deciding kidney procurement.

In addition, whether the decision for kidney procurement based on CT volumetry was actually helpful to secure better kidney function for LKDs was investigated. After donor nephrectomy, there is a 30–35% early reduction in GFR instead of 50% reduction due to functional adaptation by hyperfiltration of the remaining kidney^21,22^. To predict the compensation of the remaining kidney, Okumura et al*.* proposed the compensation prediction score (CPS) which included age, sex, history of hypertension, and remaining kidney volume indexed to body weight^23^. In addition, Lee et al. demonstrated positive significant correlation between SKF(DTPA%) and SKF(Vol%) with eGFR change during 6 months after donation^24^. In most previous reports, the split kidney volume from CT volumetry was suggested to indirectly play a role in terms of functional recovery, however, direct comparison of CT volumetry versus nuclear renography are scarce. Therefore, we focused on analyzing the predictive value of the two modalities over time. A linear fixed-effect model including age, sex, and BMI as covariables was used and showed CT volumetric measure of baseline remaining kidney function tended to be associated with higher functional recovery after donation.

There are several explanations for these results. Kidney volume correlates with the number of functioning nephrons^25^ and functional nephron mass contributes to the GFR. Hypertrophy of the remaining kidney is observed in kidney donors^26^ due to the response of nephrons to hyperfiltration after donation. Furthermore, preoperative volume of the normal kidney reportedly is an independent predictor for postoperative kidney function in radical nephrectomy^27^, similar to donor nephrectomy in LDKT. Based on these findings, we conclude that although nuclear renography can accurately estimate SKF at baseline and significantly correlates with post-donation eGFR, CT volumetric measure of SKF provides a more reliable prediction of eGFR after uninephrectomy because it reflects functional reservoir of the remaining kidney.

The current study had several limitations. Selection bias is inevitable due to the single center, retrospective, and observational nature of the study design. However, to the best of our knowledge, this is the largest series of data reported to date. Regarding study design, our transplantation center performs both CT volumetry and nuclear renography as part of donor candidate evaluation, which allowed a sufficient number of subjects for the analyses of discordant kidneys. However, in most other studies, nuclear renography was performed only if there was a definite volume difference^10,28^. Another limitation is that long-term outcome of the remaining kidney could not be determined because routine follow-up for the kidney donor was limited to 6 months after kidney donation. However, in previous studies, donation of the kidney with better function was suggested to result in adverse kidney outcome, and less initial decline in baseline eGFR after donation may be associated with better functional recovery^29,30^. These findings indicate that preservation of the kidney with better function in LKD is important and affects the long-term kidney outcome. Therefore, despite the limitations, CT volumetry was shown to provide more reliable data for functional reserve of the remaining kidney in the long-term. Lastly, whole kidney volume and not selective cortical volume was measured, which reportedly is a better non-invasive measure of functional nephron mass than whole kidney volume^31–33^. Therefore, further investigations are required to clarify the clinical significance of kidney cortical volume.

In conclusion, CT volumetry is a good imaging modality to assess SKF and outperformed nuclear renography for predicting kidney function after donation in the total cohort group and this superiority persisted when CT volumetry and nuclear renography showed discordance. Therefore, CT volumetry may be more reliable than nuclear renography for procurement strategy especially when both methods show conflicting results.

## Methods

### Patients

**Figure 3:**
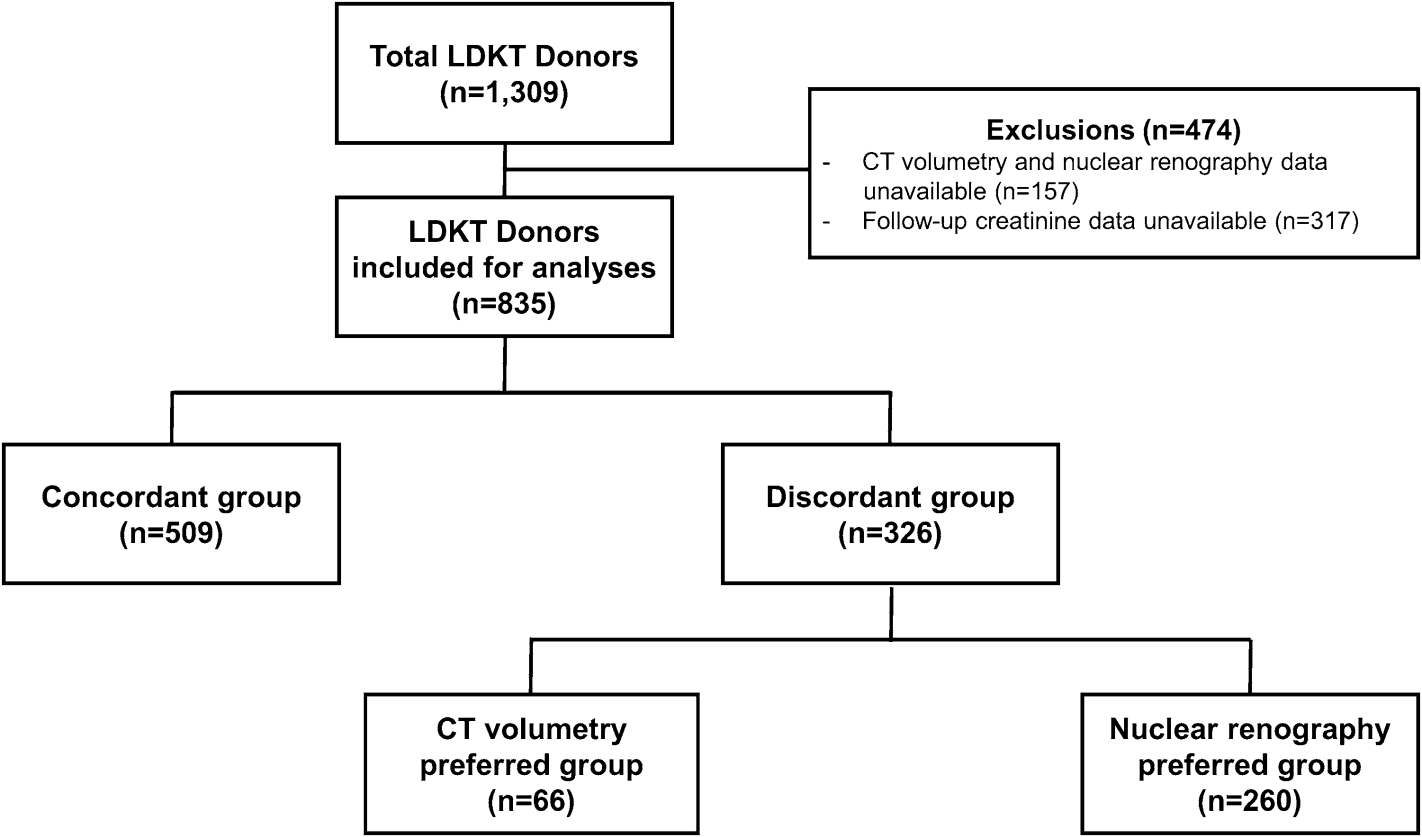
Flow chart showing donor selection in this study. *CT* computed tomography, *LDKT* living donor kidney transplantation.

In the present study, 1309 cases of kidney donors who underwent donor nephrectomies at Seoul St. Mary’s Hospital between January 2005 and April 2020 were retrospectively reviewed. We excluded 474 patients due to lack of essential data for analyses (99mTc-DTPA scintillation camera renography, CT volumetry, or follow-up creatinine after kidney donation), and 835 living kidney transplant donors were included in the final analysis (Fig. 3). This study was performed in accordance with the Declaration of Helsinki and the Declaration of Istanbul. This study was approved by the institutional review board of the Catholic University of Korea (KC20RISI1008). The requirement for informed consent from participants was waived by the institutional review board of the Catholic University of Korea due to the retrospective study design and non-invasive procedures.

### Split kidney volume and kidney function measurement

All included kidney donors were evaluated using CT and 99mTc-DTPA scintillation camera renography before kidney donation.

#### CT volumetry

Living kidney donor candidates were evaluated using CT scanners (Somatom Definition/Somatom Definition AS + , Siemens Healthcare, Erlangen, Germany; Discovery CT750 HD, GE Healthcare, Milwaukee, WI, USA; Somatom Force, Siemens Healthcare). All examinations were performed in the craniocaudal direction during suspended respiration with a 0.75 mm slice thickness, 120 kVp tube voltage, and 210 mA tube current. CT estimate of kidney length was defined as the maximum longitudinal length in the coronal section, and kidney width and thickness were measured at the largest transverse section. Contrast-enhanced CT acquisitions were obtained using 120 mL of iodinated contrast media (Iomeron; Bracco, Milan, Italy) at a rate of 4 mL/s.

Kidney volume was measured from the parenchymal phase of contrast-enhanced CT scans obtained 2 min after contrast administration. Kidney volume was estimated using the voxel-count method in which the sum of areas from each slice is multiplied by section thickness. All patient imaging data were transferred to a dedicated workstation (Aquarius 3D Workstation; TeraRecon, Foster City, CA, USA). Axial CT images with 0.75-mm slice thickness were transferred to the imaging workstation. Manual segmentation of the kidney was performed by a radiology technician on axial image and the volume of the kidney was calculated. Radiologic estimate of total kidney volume was expressed as the sum of values for the left and right kidneys.

#### 99mTc-DTPA scintillation camera renography

SKF of the LKDs was evaluated using 99mTc-DTPA scintillation camera renography. Before the examination, 99mTc-DTPA was drawn into the syringe. The pre-syringe and post-syringe counts (KcPm units) were measured. From the value of pre-syringe counts and post-syringe counts, the net injected counts were measured. With the help of gamma camera (E.Cam, Siemens Healthcare), the patient was placed on the imaging bed and the gamma camera detector was placed in a suitable position. The gamma rays emitted from the kidneys of the donors were counted by the detector of the gamma camera and the display unit showed the curve of time versus counts per minute, split kidney uptake, SKF, GFR, kidney depth, and time of maximum counts for left and right kidney using the automated software (syngo MI Applications VA46C, Siemens Healthcare).

### Clinical, laboratory data, and parameter calculations

Demographic and clinical data including age at kidney donation, sex, body mass index, donor’s relationship to recipient, and donated kidney laterality were collected. Laboratory data of serum creatinine at baseline, 1 month, 6 months, and any time point > 1 year after kidney donation were obtained. Based on serum creatinine at each time point, eGFR was calculated using the Chronic Kidney Disease-Epidemiology Collaboration (CKD-EPI) equation^18^^,^^34^. BSA was calculated using the formula of Dubois and Dubois^35^ and both kidney volume and mGFR were indexed to BSA.

Vol% is a percentage of split kidney volume of the total kidney volume. DTPA% is a percentage of split kidney mGFR of the total mGFR that came from both kidneys. Using Vol% and DTPA%, SKF (mL/min/1.73 m^2^) was calculated and expressed as SKF(Vol%) and SKF(DTPA%). SKF(Vol%) and SKF(DTPA%) were calculated as follows:

$$ SKF(Vol\% ) = \frac{eGFR \times Vol\% }{{100}}\;\;\;\;\;\;\;SKF(DTPA\% ) = \frac{eGFR \times DTPA\% }{{100}}. $$

The kidney donors were divided into concordant and discordant groups. A 50% cut-off point was used to define concordance and discordance. Concordance was defined when the better functioning kidney (DTPA% > 50%) had a larger volume (Vol% > 50%) and the worse functioning kidney (DTPA% < 50%) had a smaller volume (Vol% < 50%) in the donor. Discordance was defined when the better functioning kidney (DTPA% > 50%) had a smaller volume (Vol% < 50%) and the worse functioning kidney (DTPA% < 50%) had a larger volume (Vol% > 50%) in the donor.

### Statistical analysis

Descriptive statistics were expressed as mean ± SD or frequency (percentage) depending on the data type. Categorical variables were analyzed using Pearson’s chi-squared test. For continuous variables, means were compared using Student’s t-test. Pearson’s correlation was used to determine the correlation between quantitative datasets. Comparison of volume and mGFR between right and left kidneys was performed using paired t-tests. The degree of correlation between Vol% was assessed based on CT volumetry and DTPA% on nuclear renography. Bland–Altman plot was drawn and ICC (2,1) was calculated to show agreement between the two methods. Correlation between remaining kidney SKF (Vol%) and eGFR after kidney donation was assessed. The same analyses were performed with the remaining kidney SKF (DTPA%). The association of postoperative eGFR with pre-donation factors was explored using multivariable linear regression. Johnson's relative weights analysis was used to estimate the relative importance of each potential determinant and the results were expressed as percentage contribution to multiple *R*^36^. A linear mixed-effect model was used to assess kidney function recovery after kidney donation (SKF at baseline, 1 month, 6 months, and > 1 year) between CT volumetry preferred group and nuclear renography preferred group. Estimated marginal means from the model was expressed as Estimated marginal mean ± standard error (SE). The model included a random intercept for individuals and was adjusted for potential cofounders (age, sex, and BMI). The interaction of time points and group was tested to explore if kidney function changed differently over time between the two groups. In all analyses, p < 0.05 (two-tailed) was considered to indicate statistical significance. All statistical analyses were performed with SPSS package (Version 24; SPSS, Inc., Chicago, IL, USA). In addition, all graphs were generated using Prism software (GraphPad, San Diego, CA, USA).

## Data Availability

The datasets generated during and/or analyzed during the current study are available from the corresponding author on reasonable request.
